# Privacy Risk Perception of Online Medical Community Users Based on Deep Neural Network

**DOI:** 10.3389/fpsyg.2022.914164

**Published:** 2022-06-15

**Authors:** Pei Yin, Jun Zhang, Han Yan, Jun Zhao, Jing Wang, Chunmei Liang

**Affiliations:** Business School, University of Shanghai for Science and Technology, Shanghai, China

**Keywords:** deep neural network, online medical community, highly complex and nonlinear data, evaluation model, risk perception

## Abstract

This paper studies the privacy risk perception of online medical community users based on deep neural network. Firstly, this paper introduces privacy protection based on deep neural network and users’ privacy risk perception in online medical community. Then, using the fuzzy neural network to deal with highly complex and nonlinear data, we can better obtain the accurate evaluation value, and use the improved gravity search optimization algorithm to optimize the fuzzy neural network evaluation model and improve the convergence puzzle of the model. Finally, using the experimental method of questionnaire survey, and the questionnaire is composed of three parts. The first part investigates the basic personal information of the subjects, including gender, age, educational background, physical condition, physical examination frequency, Internet use experience, long-term residence, etc.; The second part is the measurement items of each variable in the theoretical model, including nine variables: service quality, personalized service, reciprocal norms, result expectation, material reward, perceived risk, trust in doctors, trust in websites, and willingness to disclose health privacy information. The experimental results show that the correlation coefficient between the interaction items of personalized service and reciprocal norms on material reward is positive (β = 0.072, *P* < 0.01), and the correlation coefficient between sexual service and material reward was positive (β = 0.202, *P* < 0.01), then reciprocal norms positively regulate the relationship between personalized service and material reward.

## Introduction

**FIGURE 1:**
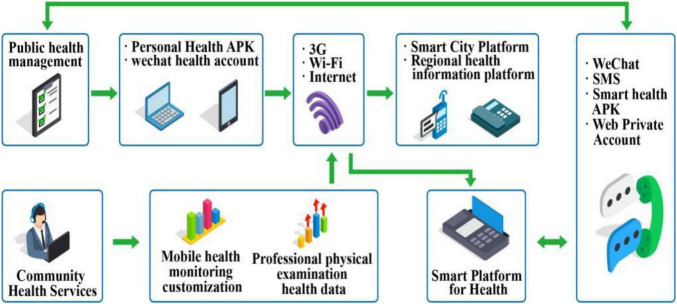
Online community medical service.

The advent and rapid development of the network era not only brings many conveniences to contemporary people, but also comes with an important problem. With the advent of the big data era, the increasingly mature network world makes information grow everywhere. At the same time, the current social public seems to have no privacy. Powerful search engines collect and record people’s browsing information and personal information input by the medical system all the time, and face the risk of information leakage caused by network risk. In the face of the rapid development of the network, people’s personal privacy, information security, and risk perception are particularly important (Inayat et al., 2021). With the continuous development of medical undertakings (Figure 1), the online medical community has gradually developed and expanded under the blessing of the Internet. With the increasing number of users in the online medical community, the security of users’ medical privacy information is becoming worrying. The continuous enrichment of medical information content can help doctors understand the patient’s information and help patients find problems to the greatest extent, but at the same time, user privacy information security sharing and risk prevention are particularly important.

Focusing on the theme of influencing factors of online community members’ privacy information sharing behavior, scholars at home and abroad mainly carry out research from three major directions (Alyaev et al., 2021). Direction 1: user’s own factors, such as demographic characteristics, personality, habits, expected benefits, perceived usefulness, privacy concerns, etc.; Direction 2: network platform factors, such as external incentives provided by website service providers, Website Trust, privacy policies, etc.; Direction 3: social influencing factors, such as subjective norms and peer effect. However, the research results of explaining personal information disclosure from the perspective of user perception are not perfect. The existing research mainly involves the impact of trust and perceived usefulness, trust risk model, comprehensive perceived income and privacy concerns on users’ willingness to privacy disclosure. After systematically combing the relevant literature, we find that although scholars agree that trust, perceived return and perceived risk occupy a very important position in personal information disclosure, few scholars integrate these three factors into a theoretical framework to reveal the mechanism and boundary conditions of personal privacy information disclosure intention from a more comprehensive perspective (Kilcioglu et al., 2021).

## Literature Review

In the aspect of deep neural network privacy protection, the early research mainly focused on the privacy protection in the training stage of neural network. For example, Lu, L. and others proposed a privacy protection scheme for outsourcing training process of single-layer perceptron. Single layer perceptron is a simple neural network. They use the threshold function as the activation function, and use the Paillier plus homomorphic cipher scheme to realize the secure calculation of data (Lu, 2021). Duan, X. and others believe that since the additive homomorphism only supports the corresponding plaintext encryption in the ciphertext state, and the calculation of the threshold function cannot be realized, the data owner needs to decrypt the calculation when calculating the activation value, and then send the activation value to the cloud server. It is easy to know that this scheme is not safe. In each iteration, the data owner will send the activation value to the cloud server for subsequent calculation (Duan et al., 2020). Huang, Z. and others proposed a privacy protection training protocol based on BGN encryption scheme and secret sharing scheme. BGN is a double homomorphic encryption scheme. It only supports one multiplication and infinite addition operations. Therefore, the intermediate value of multiplication operation must be decrypted and re encrypted to continue the subsequent operation. In order to prevent the intermediate value from being leaked in decryption, they use secret sharing scheme to divide the intermediate value into multiple parts (Huang et al., 2020). Tu, X. and others realized the privacy protection in the prediction stage of convolutional neural network by using square function instead of activation function and hierarchical homomorphic encryption scheme; A scheme for privacy protection prediction without using cryptography technology (Tu et al., 2021). El Ghandour, M. and others believe that the user has the weight of personal data and the first layer neural network. The user locally calculates the first layer activation output, then randomly deletes some output nodes and sends the first layer output to the server. Because some output is deleted, the result is irreversible, thus ensuring the user’s privacy. At the same time, users can only get the first layer parameters of the server, and other parameters are unknown to users. Therefore, privacy protection neural network prediction is realized; Based on Paillier encryption, a privacy protection prediction scheme under dual cloud server system is proposed (El-Ghandour et al., 2021). Lee, D. W. and others believe that in their scheme, the user and Cloud B share the private key, cloud a has the model parameters, the user encrypts the eigenvector and sends it to cloud a, cloud a is responsible for calculating the dot product, and Cloud B is responsible for calculating the activation function; A privacy preserving neural network scheme based on multi-party joint learning without sharing data is designed. All users share a parameter server. Each user trains the neural network locally, and then uploads the updated gradient to the parameter server. Other users download parameters for further training. In order to prevent too much information disclosure, they use selective parameter update and differential privacy to minimize parameter disclosure; In order to avoid people or servers other than users getting parameters, Paillier homomorphic encryption scheme is used to encrypt the gradient of each update before uploading. The server can only calculate the ciphertext of the latest parameters according to the ciphertext, but the server has no decryption secret key and cannot get the parameter information, and all users share the decryption secret key (Lee et al., 2021). Lee, E. and others believe that personalized services and service quality, as platform service factors, complement each other and jointly affect the survival and development of online community platforms. Their impact on users’ willingness to share privacy is particularly important (Lee and Rhee, 2021). Research by Liang, W. and others shows that personalized services can act on users’ perceived benefits and perceived risks, and then have an impact on privacy sharing willingness (Liang et al., 2021). Sun, H. et al. Believe that in the context of online health care community, the promotion mechanism of personalized service and service quality affecting user perception has not been explored. Previous studies have shown that knowledge sharing behavior in the Internet will be strongly promoted and affected by reciprocal norms (Sun et al., 2021).

## Privacy Protection Based on Deep Neural Network

### Neural Network

(1) Perceptron

**FIGURE 2:**
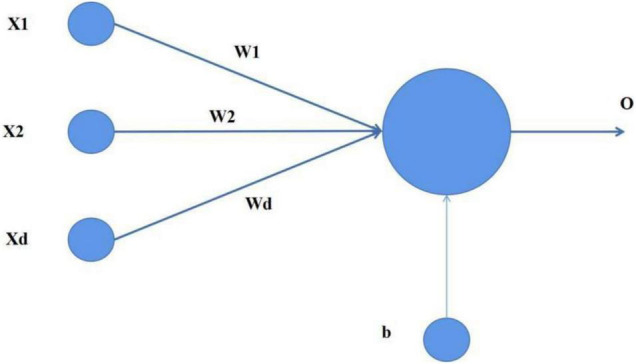
Perceptron.

Perceptron is the simplest neural network in neural networks. It is a binary linear classifier. Although its structure is simple, it can effectively deal with complex binary classification problems. As shown in Figure 2.

where (x1, x2, …, xd) is the input, (w1, w2, …, wd) is the weight, and B is the deviation. The input of the perceptron is shown in formula 1:


(1) $$y_{p}=f\,\left(\sum_{i}w_{i}\,x_{i}+b\right)=s\,i\,g\,n\,\left(\sum_{i}w_{i}\,x_{i}+b\right)$$

In the training process of perceptron, the parameter update methods include random gradient update, small batch gradient update and batch gradient update. The corresponding formulas of the three update methods are as follows:


(2) $$w_{i}=w_{i}+\Delta \,w_{i}$$

(3) b=b+Δ⁢b

The random gradient is updated as follows:


(4) $$\Delta \,w_{i}^{\left(j\right)}=\lambda \,\left(y_{i}^{\left(j\right)}-y_{p}^{\left(j\right)}\right)\,x_{i}^{\left(j\right)}$$

Small batch gradient updates are as follows:


(5) $$\Delta \,w_{i}^{\left(j\right)}=\sum_{j}^{b\,a\,t\,c\,h}\lambda \,\left(y_{i}^{\left(j\right)}-y_{p}^{\left(j\right)}\right)\,x_{i}^{\left(j\right)}$$

Batch is the batch size updated as follows:


(6) $$\Delta \,w_{i}^{\left(j\right)}=\sum_{j}^{m}\lambda \,\left(y_{i}^{\left(j\right)}-y_{p}^{\left(j\right)}\right)\,x_{i}^{\left(j\right)}$$

where m represents the number of all training samples.

(2) Convolutional neural network

Convolutional neural network is a special neural network which is good at analyzing visual images and is widely used in image and video recognition, classification and natural language processing. Convolutional neural network mainly includes convolution layer, pooling layer, activation layer and full connection layer.

Convolution layer: convolution layer is the core of convolution neural network, which is mainly used to extract features (Deng et al., 2021). The convolution layer adopts the way of local connection, and each neuron of the convolution layer is connected with only part of the neurons of the upper layer. The convolution layer often contains multiple convolution cores, which slide in all positions of the image. At each position, the convolution core and the input do dot product operation.

**FIGURE 3:**
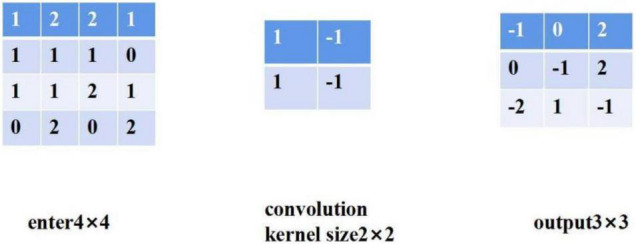
Convolution operation.


A simple convolution process is shown in Figure 3. The elements of the first row and the first column of the output are obtained by multiplying each element in the first two rows and columns of the input and the corresponding element in the convolution kernel. The formula is as follows:

(7) -1=1×1+2×(-1)+1×1+1×(-1)

Activation layer: the activation layer is generally used after the convolution layer and the full connection layer to make a nonlinear mapping of the output result of the previous layer. In convolutional neural networks, the modified linear unit function [rectified linear unit (RELU)] is often used as the activation function. RELU can make only about 50% of neurons in the network active (Song et al., 2021), effectively reduce the occurrence of over fitting, and it can effectively avoid the problems of gradient explosion and gradient disappearance. The mathematical form of the modified linear unit function is as follows:


(8) f⁢(x)=max⁡(0,x)

**FIGURE 4:**
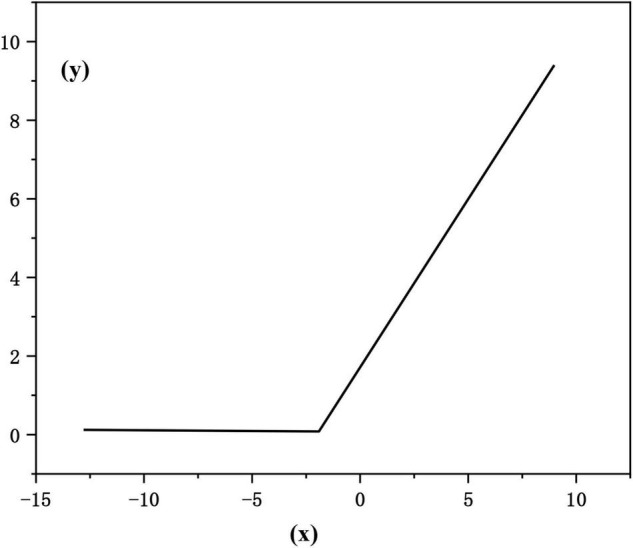
RELU function.

**FIGURE 5:**
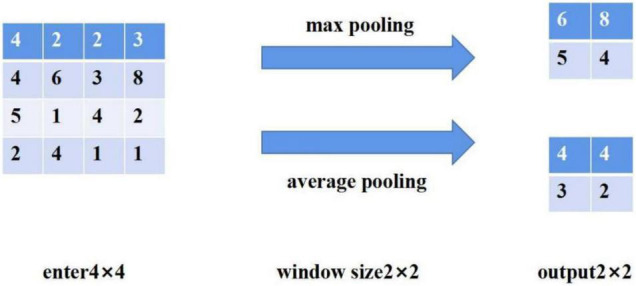
Pool operation.

The function image is shown in Figure 4:

Pooling layer: used to reduce dimension, that is, compress irrelevant information in the image, retain important image features and prevent over fitting. Common pooling methods include maximum pooling and average pooling. Maximum pooling is to take the maximum value in the pooled area, while average pooling is to take the average value of all values in the pooled area. Figure 5 is a simple example of maximum pooling and average pooling (Lee and Park, 2021).

**FIGURE 6:**
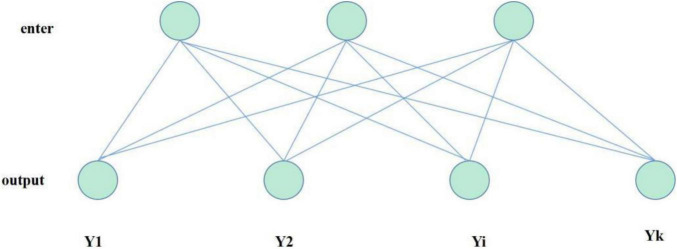
Full connection operation.


Full connection layer: use the extracted features for classification or regression. Each neuron in the whole connecting layer is connected to each neuron in the previous layer. As shown in Figure 6, it is assumed that layer *l-1* contains d neurons, each neuron is marked as (l1), *j*_*x*_, layer l contains k neurons, each neuron is marked as f, the weight between the i-th neuron of layer l and the j-th neuron of layer *l-1* is expressed as $w_{l,i\,j}^{f\,c}$, and the output calculation formula of the full connection layer is:

(9) $$y_{l,i}=\left(\sum_{j}x_{\left(l-1\right),j}\times w_{l,i\,j}^{f\,c}\right)+b_{l,i}^{f\,c}$$

In convolutional neural networks, softmax function is usually used to calculate the probability value of the output result of the last layer of the network (that is, the probability value belonging to each class). In the prediction process, the deletion of this function will not affect the classification result (Cheng et al., 2021).

### Privacy Information Retrieval

Privacy information retrieval is a two-party agreement. The first participant a can retrieve the m-th information from the second party’s database, but the second participant B cannot know which information a retrieved. A simple privacy information retrieval protocol based on homomorphic encryption.

### Dot Product Agreement for User Privacy Information Protection

Privacy preserving dot product protocol is a two-party protocol for privacy calculation of two vector dot products (Basheer et al., 2021).

According to the additive homomorphism of Paillier encryption, the following formula can be obtained:


$$\prod_{\text{i}=1}^{\text{d}}{\left[\left[a_{i}\right]\right]}^{b_{i}}=\left[\left[\sum_{i=1}^{d}a_{i}\,b_{i}\right]\right]=\left[\left[a_{1}\,b_{1}+a_{2}\,b_{2}+\ldots +a_{d}\,b_{d}\right]\right]$$

(10) $$=\left[\left[\vec{a}\times \vec{b}\right]\right]$$

It mainly introduces some modules used in subsequent privacy protection protocols, including privacy protection dot product protocol, privacy protection comparison protocol, privacy protection Max protocol, and privacy protection argmax protocol. These protocols can realize privacy protection computing under the semi honest model. We give the correctness and security proof of these protocols based on secure multi-party computing and module sequence composition (Chen et al., 2021).

### Correctness and Security of the Agreement

To prove that the protocol is correct, we need to prove that the gradient update result of each iteration is correct.

The gradient update formula of small batch is:


(11) $$w_{i}=w_{i}+\Delta \,w_{i}$$

Small batch gradient updates are as follows:


$$\Delta \,w_{i}^{\left(j\right)}=\sum_{j}^{b\,a\,t\,c\,h}\lambda \,\left(y_{i}^{\left(j\right)}-y_{p}^{\left(j\right)}\right)\,x_{i}^{\left(j\right)}$$

$$=\lambda \,\left(\sum_{j}^{b\,a\,t\,c\,h}\left(y_{i}^{\left(j\right)}\,x_{i}^{\left(j\right)}-y_{p}^{\left(j\right)}\,x_{i}^{\left(j\right)}\right)\right)$$

(12) $$=\lambda \,\left(\sum_{j}^{b\,a\,t\,c\,h}\left(y_{i}^{\left(j\right)}\,x_{i}^{\left(j\right)}\right)-\sum_{j}^{b\,a\,t\,c\,h}\left(y_{p}^{\left(j\right)}\,x_{i}^{\left(j\right)}\right)\right)$$

To get the predicted value of training data in each iteration, deblinding is required, that is:


(13) $${\left\{\left[\left[y_{p}^{\left(j\right)}\right]\right]\right\}}_{j=1}^{b\,a\,t\,c\,h}\leftarrow {\left\{{\left[\left[y_{p}^{\left(j\right)}\right]\right]}^{s\,i\,g\,n\,\left(\alpha \right)}\right\}}_{j=1}^{b\,a\,t\,c\,h}$$

(14) $${\left\{\left[\left[\vec{x^{\left(j\right)}}\times y_{p}^{\left(j\right)}\right]\right]\right\}}_{j=1}^{b\,a\,t\,c\,h}={\left\{{\left[\left[\vec{x^{\left(j\right)}}\times y_{p}^{\left(j\right)}\right]\right]}^{s\,i\,g\,n\,\left(\alpha \right)}\right\}}_{j=1}^{b\,a\,t\,c\,h}$$

Substituting into formula 12, we can get the gradient:


(15) $$\Delta \,\vec{w}={\left[\left[\lambda \,\sum_{j}^{b\,a\,t\,c\,h}\left(y_{t}^{\left(j\right)}-y_{p}^{\left(j\right)}\right)\times x^{\left(j\right)}\right]\right]}^{\left(\frac{1}{b\,a\,t\,c\,h}\right)}$$

(16) $$\Delta \,b={\left[\left[\lambda \,\sum_{j}^{b\,a\,t\,c\,h}\left(y_{t}^{\left(j\right)}-y_{p}^{\left(j\right)}\right)\times x^{\left(j\right)}\right]\right]}^{\left(\frac{1}{b\,a\,t\,c\,h}\right)}$$

According to formula (14)–(15), the gradient can be updated correctly by using additive homomorphism.

### Experimental Results

**FIGURE 7:**
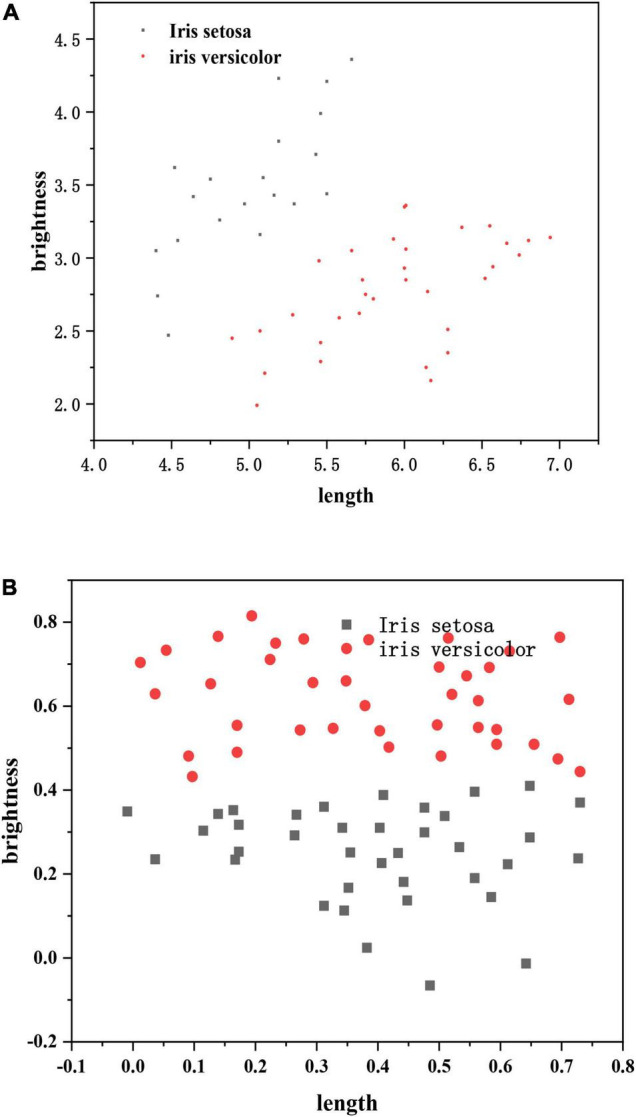
Data distribution. **(A)** IRIS data set and **(B)** LOWBWT data set.

In order to test the feasibility of the perceptron privacy protection training protocol proposed in this chapter, this paper uses a computer with processor 4-core3.0GHzIntelCorei5-7400 and memory of 8GBRAM to complete the experiment. For the training of perceptron, we use Python language under 64 bit windows operating system and implement it based on Tensorflow framework. We use the partial homomorphic encryption library phe1.4.0 of Python 3 to realize Paillier encryption, and use the scientific computing library numpy1.14.5 for array or matrix operation. We use two real data sets IRIS and LOWBWT to test the accuracy and efficiency of this privacy protection training protocol. Iris data set comes from UCI machine learning library (Sengly et al., 2021). In order to better understand the distribution of data sets, we use the two characteristic attributes of IRIS and LOWBWT data sets to visualize the data (as shown in Figure 7).

Training on iris dataset and LOWBWT dataset. The time required to complete an iteration (minimum batch gradient update) by changing the batch size to 5, 15, 25 is shown in Table 1. It can be seen from Table 1 that with the increase of characteristic attributes of training data, the time will also increase. With the increase of batch size, the time will also increase. It is easy to get. The time required for one iteration is related to the number of data feature attributes and batch size.

**TABLE 1:** Time taken to update parameters once (s).

Batch size	5	15	25
IRIS	1.117207	3.004752	5.016047
LOWBWT	1.402311	3.529103	5.713865

For the prediction of perceptron, in order to reduce the computing overhead and achieve efficient prediction, this paper uses the cloud platform, which can provide powerful storage and computing power (Guzman, 2020). With the rapid development of cloud computing, outsourcing computing, and outsourcing storage has become a trend. Storage and computing with the help of cloud platform can effectively reduce the burden of user management and processing data, but if the data is simply transmitted to the cloud, it is bound to cause privacy disclosure. Aiming at the problem of perceptron prediction, a cloud assisted perceptron privacy protection prediction scheme is proposed, which can effectively protect the privacy of input features and model parameters in the prediction process.

## Online Medical Community Users’ Privacy Risk Perception and Protection

### Study Design

(1) Questionnaire design

The questionnaire consists of three parts. The first part investigates the basic personal information of the subjects, including gender, age, educational background, physical condition, frequency of physical examination, Internet use experience, long-term residence, etc.; The second part is the measurement items of each variable in the theoretical model, including nine variables: service quality, personalized service, reciprocal norms, result expectation, material reward, perceived risk, trust in doctors, trust in websites, and willingness to disclose health privacy information. In order to ensure the reliability and validity of the measurement, the maturity scale in foreign research is used in this study, the back translation is used to ensure the accuracy of the questionnaire, and some items are modified in combination with the development status of medical websites in China. Among them, the measurement items of perceived risk are selected based on the scales used in Roselius, Featherman, etc., and Cases’ research. The measurement items of reciprocal norms, material rewards, outcome expectations and willingness to disclose personal health information are derived from the maturity scale in the research of Chiu and others. And Kankanhalli and others personalized service and service quality come from the research of Xu and others and Rodriguez and others. The trust in doctors and websites comes from the research of Kankanhalli and others. There are 32 items in the questionnaire. All items are scored by Likert five point scale. The subjects choose between 1 and 5. 1 means very disagree and 5 means very agree.

(2) Sample selection and data collection

Since the main target users of the online medical website are people with various diseases, and the middle-aged people over 40 are the groups with frequent diseases, we choose them as our main respondents. We distributed and collected questionnaires in wechat circle of friends through the questionnaire star network platform. In this study, a total of 300 questionnaires were distributed and 267 were recovered, with a questionnaire recovery rate of 89%. After screening, 264 valid questionnaires were obtained, with an effective questionnaire rate of 98.87%. From the perspective of sample size, it meets the needs of sample size and achieves the purpose of stabilizing samples (Wendy, 2018).

According to the results of descriptive statistical analysis, we found that men accounted for 41.7% and women accounted for 58.3%, and the gender ratio was basically balanced; 9.5% were aged 20 years and below, 39% were aged 21–30 years, 10.2% were aged 31.40 years, 32.6% were aged 41–50 years, 7.6% were aged 51–60 years, and 1.1% were aged 61 years or above, indicating that the main research group in this study was people aged 20–50 years; The educational background distribution is relatively uniform, among which 91.7% of the subjects have education above senior high school (Bhagavathula et al., 2022); The surveyed users from second tier cities (provincial capitals and municipalities directly under the central government) and ordinary cities and towns accounted for 90.2% of the total sample, while the samples from first tier cities (Beijing, Shanghai, Guangzhou, Shenzhen) and rural areas accounted for only 9.8% of the total sample; From the physical condition of the subjects, only 24.2% of the subjects were disease-free, and 75.7% of the subjects had different degrees of disease or sub-health, which proved that the research object of this study met the needs of the research situation. In terms of physical examination frequency, 94.7% of the subjects had almost no physical examination and one-year physical examination; At the same time, the data also shows that people with more than 3 years of experience in using health websites account for 74.2% of the sample size, indicating that most of the subjects in this study have long-term experience in using health websites, can better understand the research situation and fill in the questionnaire according to their own experience.

### Empirical Test

(1) Reliability and validity test

In order to test the reliability of the scale in this study, we used Cronbach’s α coefficient, combined reliability (CR) and mean variance extraction (AVE) to measure. According to previous studies, generally, if the value of Cronbach’s α coefficient is greater than 0.7, it can be considered that the observed variable has good reliability to the latent variable. According to the calculation results in Table 2, the Cronbach’s α coefficient of each variable is between 0.848 and 0.928, so the scale in this study has good reliability. In order to further test the reliability and validity of the scale, this study measured the combined reliability (CR) and mean variance extraction (AVE). and the combined reliability value is between 0.854 and 0.930, both of which are greater than the standard of 0.700; At the same time, the ave of all variables is between 0.506 and 0.784, which is greater than the standard of 0.500, indicating that the convergence validity of the scale in this study is good; The factor load (FL) value of each item in the measurement variable is greater than 0.5, and each measurement item can better represent the measurement variable, indicating that the scale has good convergence validity. In addition, this study uses the method of comparing the square root of ave value with the absolute value of correlation coefficient to test the discriminant validity. The square root of ave of each variable is greater than 0.600, indicating that the difference between the measurement items of different variables is large and the discriminant validity is high. In order to test the discrimination validity between the key variables “service quality,” “personalized service,” “reciprocal norms,” “result expectation,” “material reward,” “perceived risk,” “trust in doctors,” “trust in websites” and “willingness to disclose health privacy information,” as well as the corresponding measurement parameters of each measurement scale. In this study, AMOS20.0 was used for confirmatory factor analysis of key variables, and the nine factor model was compared with eight factors model, seven factor model and six factor model. The results show that the nine factor model is in good agreement, as follows:

**TABLE 2:** Non standardized coefficient of adjustment effect test.

Variable	Perceived risk				Result expectation			
	Model 1		Model 2		Model 3		Model 4	
Predictor	Estimate	SE	Estimate	SE	Estimate	SE	Estimate	SE
Gender	0.148	0.123	0.099	0.122	0.008	0.089	0.024	0.091
Age	0.067	0.068	0.057	0.066	–0.049	0.049	–0.030	0.050
education	0.208	0.067	0.214	0.066	0.078	0.049	0.031	0.035
Physical condition	–0.008	0.042	–0.148	0.0365	0.049	0.033	0.031	–0.193
Physical examination frequency	–0.051	0.067	0.035	–0.012	0.068	0.049	0.035	–0.056
Internet use experience	–0.068	0.067	–0.102	0.066	–0.021	0.049	–0.001	0.049
Long term residence	0.046	0.099	0.043	0.097	0.063	0.072	0.052	0.073


$$\chi^{2}=940.631$$

d⁢f=428

$$\chi^{2}/d\,f=2.198$$

N⁢F⁢I=0.870

I⁢F⁢I=0.925

T⁢L⁢I=0.912

C⁢F⁢I=0.924

R⁢M⁢R=0.052

(17) R⁢M⁢S⁢E⁢A=0.067

Moreover, this model is significantly better than the other eight models, indicating that the measurement of the scale in this study has good discriminant validity.

(2) Hypothesis testing

This paper uses AMOS20.0 to test the main effect and its mediating effect of the structural model, that is, to confirm whether the hypothesized relationship between latent variables in the theoretical model constructed in this study is established. First, it is tested whether the theoretical model can be completely matched with the survey data. The fitting results of the model are as follows:


$$\chi^{2}=1136.435$$

d⁢f=363

$$\chi^{2}/d\,f=3.131$$

N⁢F⁢I=0.826

I⁢F⁢I=0.874

T⁢L⁢I=0.859

C⁢F⁢I=0.874

R⁢M⁢R=0.118

(18) R⁢M⁢S⁢E⁢A=0.090

**FIGURE 8:**
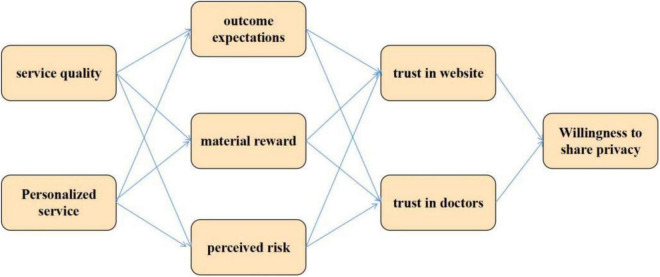
Non standardized path coefficient diagram.

From the goodness of fit index, the result is ideal, which shows that the overall theoretical model has good adaptability. Among them, the relationship coefficient between material reward and personalized service is 0.064, *P* = 0.414, which is not significant and has a positive relationship. It is assumed that H1 is not tenable. The relationship coefficient between perceived risk and service quality is −0.003, *P* = 0.974, which is not significant and has a negative relationship. It is assumed that H6 is not tenable. Other assumptions are true, as shown in Figure 8. The path coefficient reflects the strength and direction of the influence of independent variables on dependent variables in the theoretical model. The larger the path coefficient, the greater the influence. When the path coefficient is positive, it indicates that the influence of the independent variable on the dependent variable is positive, and vice versa.

Plus software is famous for its powerful data calculation function. It can present the data results in a concise way and better estimate the indicators related to the regulation effect. Therefore, we use this software package to calculate the data. According to the test rules of regulatory effect in previous studies, the regulatory effect is established only if the correlation coefficient of the interaction term to the dependent variable is significant. From the data results in Table 2, in model 1, the correlation coefficient between the interaction term of service quality and reciprocity specification for perceived risk is negative and significant (β = −0.093, *P* < 0.05). The correlation coefficient between service quality and perceived risk is negative (β = −0.016, *P* > 0.1), indicating that reciprocal norms positively regulate the relationship between service quality and perceived risk. In mode 2, the correlation coefficient between the interaction items of personalized services and reciprocal norms for perceived risk is negative and significant (β = −0.143, *P* < 0.01), and the correlation coefficient between personalized service and perceived risk was positive (β = 0.030, *P* > 0.1), indicating that reciprocal norms negatively regulate the relationship between personalized services and perceived risk. In mode 3, the correlation coefficient between service quality and the expected result of the interaction term of reciprocity specification (β = −0.002, *P* > 0.1) is negative and not significant, then the regulatory effect of reciprocity Specification between service quality and result expectation is not tenable. The interaction coefficient of mode 4 is not significant in the expectation of personalized service (β = 0.031, *P* > 0.1), indicating that the regulatory effect of reciprocity norms between personalized service and result expectation is not tenable. In mode 5, the correlation coefficient between the interaction term of service quality and reciprocal norms and material reward is positive and significant (β = 0.077, *P* < 0.05). The correlation coefficient between service quality and material reward is positive and significant (β = 0.283, *P* < 0.01), then reciprocal norms have a significant positive regulatory effect between service quality and material reward. In mode 6, the correlation coefficient between the interaction term of personalized service and reciprocal norms and material rewards is positive (β = 0.073, *P* < 0.1), and the correlation coefficient between personalized service and material reward is positive (β = 0.203, *P* < 0.01), then reciprocal norms positively regulate the relationship between personalized service and material reward. Based on the above analysis of the data results, it is assumed that H15, H16, H19, and H20 are true, H17 and H18 are not true.

### Research Conclusion

Firstly, previous studies have discussed the impact of personalized services on privacy sharing, but ignored the important role of service quality. This study confirms the positive impact of service quality and personalized service on users’ willingness to share health information privacy, makes up for the neglect of this issue in previous studies, develops users’ privacy computing theory, and provides a new perspective for the research of health privacy sharing. Secondly, this study confirmed that outcome expectations and material rewards as perceived benefits will positively promote users’ trust in websites and doctors, and then lead to the generation of privacy sharing intention. This study introduced outcome expectations and material rewards into the theoretical framework of perceived benefits from a new perspective, and confirmed their positive impact on trust. Thirdly, this study reveals the internal mechanism of users’ willingness to share privacy under the influence of service quality and personalized service. In the Chinese scenario, we support the privacy computing theory, that is, users generate trust after weighing perceived benefits (result expectation, material reward) and perceived risks, and trust is one of the preconditions for generating privacy sharing intention, which shows that the two are the bridge between the characteristics of network environment and users’ healthy privacy sharing behavior. More importantly, this study proposes and verifies that personalized service and service quality reveal the psychological process mechanism of users’ willingness to share health and privacy information through the intermediary effect of perceived benefits (result expectation, material reward) and perceived risk. Finally, this study verifies that user behavior is the result of the interaction between personal factors and network situational factors, and explains the reasons for the differences in users’ willingness to share privacy under the same scenario. Under the function of strong reciprocity norms, service quality and personalized service can gradually weaken users’ perceived risk, so as to indirectly weaken the negative effect of perceived risk on users’ trust. In addition, at this time, service quality and personalized service can indirectly stimulate stronger user trust by enhancing users’ expectations for material rewards of the website. Indeed, trust is the basis of health privacy information sharing. Users’ trust in the website greatly promotes the sharing of personal health privacy information. Therefore, the strong reciprocal norms established by the website are the basis of users’ willingness to share health information. However, unfortunately, this study does not confirm the moderating effect of reciprocity norms between service quality and outcome expectation, personalized service and outcome expectation. We believe that this is because the reciprocity norms established by the website can ensure that users can obtain corresponding benefits from their efforts on the website, so reciprocity norms cannot enhance the impact of service quality and personalized service on outcome expectation.

According to the research results of this paper: (1) Personalized service and service quality have an indirect impact on users’ willingness to share privacy. Therefore, website managers should comprehensively consider the innovation of service model in website construction. It is generally believed that personalized services can attract users’ attention, retain users, and then share personal health and privacy information. However, our research found that personalized services may enhance users’ perceived risk, while service quality can reduce users’ perceived risk to a certain extent, even if the impact is not significant. Personalized service and service quality, as the core indicators to promote user privacy sharing, are indispensable. This requires website managers to find an appropriate balance in the establishment and management of websites, integrate personalized services and service quality into a system, develop in a symbiotic way, and finally form a website operation atmosphere with dual attributes. Only in this way can the medical and health website really gain vitality, otherwise it will only make the medical and health website take care of one thing and lose the other. Therefore, on the one hand, we need to pay attention to the needs of users and provide personalized services. On the other hand, we also need to check the service quality of medical websites, and introduce high-quality medical information and high-level and high-quality doctors into the medical service platform. We should not only serve patients pertinently, but also help patients solve the problem of seeking medical advice, so that patients can actively share personal health privacy information.

(2) Website operators must make it clear that users’ trust in the website and doctors on the website is the premise of health and privacy information sharing willingness, and try their best to overcome the difficulty of users’ low trust in the network. As perceived benefits, outcome expectations and material rewards can act on trust together with perceived risk and affect users’ willingness to share health privacy information. Through the publicity of successful cases of the website, website managers can enhance users’ expectations for the results of obtaining efficient information on the website. At the same time, website operators can also attract users by providing material rewards in the form of coupons, lucky draw, point exchange and free trial, so as to directly enhance their trust in the website. More importantly, website operators must understand that the openness of the network environment leads to the frequent disclosure of users’ privacy, which subjectively increases users’ perceived risk and creates trust barriers. Providing high-quality personalized services can be the first choice for operators. The purpose of this is to make users’ medical needs truly satisfied on the medical website, produce an immersive experience, and then spontaneously and actively share private information about personal health.

## Conclusion

The study found that reciprocal norms can significantly reduce perceived risks and enhance perceived benefits. Website operators should establish a set of game rules that can benefit both sides in the process of interaction according to the interaction mode between doctors and patients on the website. For example, formulate norms to allow patients to recharge, and pay doctors on the website for each consultation on the condition. At the same time, patients score after the doctor’s service. This score can be used as an evaluation index of doctor’s service and linked to the doctor’s income. Through this game mode of mutual benefit, it can restrain both doctors and users. It cannot only stabilize the doctors and patients who use the website, but also increase the trust of patients and doctors in the website as a medium, resulting in more active sharing of health privacy.

This study has obtained some useful results, but also has some limitations, which need to be improved by follow-up research. Firstly, most of the measurement indicators used in this paper come from relevant foreign studies. Although the data results of each variable show that the reliability and validity of the scale used in this study are good, if we can develop a scale suitable for Chinese situation, it will make the measurement of variables more accurate. Secondly, this paper discusses the impact of service quality and personalized service on users’ willingness to share health information. In fact, there are still many boundary conditions in this process. In addition to the consideration of website level and individual perception level, relevant factors such as individual personality and cultural background also deserve attention.

## Data Availability Statement

The original contributions presented in the study are included in the article/supplementary material, further inquiries can be directed to the corresponding author.

## Author Contributions

PY and JZ: writing and static analysis of data. HY, JZ, and JW: experimental operation. CL: check. All authors contributed to the article and approved the submitted version.

## Conflict of Interest

The authors declare that the research was conducted in the absence of any commercial or financial relationships that could be construed as a potential conflict of interest.

## Publisher’s Note

All claims expressed in this article are solely those of the authors and do not necessarily represent those of their affiliated organizations, or those of the publisher, the editors and the reviewers. Any product that may be evaluated in this article, or claim that may be made by its manufacturer, is not guaranteed or endorsed by the publisher.
